# Predicting Hand Washing and Sleep Hygiene Behaviors among College Students: Test of an Integrated Social-Cognition Model

**DOI:** 10.3390/ijerph17041209

**Published:** 2020-02-13

**Authors:** Chun-Qing Zhang, Rongyu Fang, Ru Zhang, Martin S. Hagger, Kyra Hamilton

**Affiliations:** 1 Department of Sport and Physical Education, Hong Kong Baptist University, Hong Kong; 2 School of Psychology, Curtin University, Perth, WA 6102, Australia; 3 Department of Hygiene and Health Management, Qujing Medical College, Qujing 655011, China; 4 Department of Sports Science & Physical Education, The Chinese University of Hong Kong, Hong Kong; ruzhang@cuhk.edu.hk; 5 Psychological Sciences, University of California, Merced, CA 95343, USA; mhagger@ucmerced.edu; 6 Faculty of Sport and Health Sciences, University of Jyväskylä, 40014 Jyväskylä, Finland; 7 School of Applied Psychology, Griffith University, Brisbane, QLD 4122, Australia; kyra.hamilton@griffith.edu.au

**Keywords:** hand washing, Health Action Process Approach, health behavior, sleep hygiene, Theory of Planned Behavior, young people

## Abstract

*Objective:* Hand washing and sleep hygiene are two important health behaviors. The purpose of the current study was to identify the motivational and volitional antecedents of college students’ hand washing and sleep hygiene behaviors based on an integrated model of behavior that combined social-cognition constructs from the Theory of Planned Behavior (TPB) and Health Action Process Approach (HAPA). *Methods:* Using a prospective design, college students (*N* = 1106) completed a survey assessing the motivational constructs of action self-efficacy, attitudes, subjective norm, perceived behavioral control, intentions, and behaviors of hand washing and sleep hygiene at Time 1. Demographic variables were also collected. One month later, at Time 2, college students (*N* = 524) self-reported on their volitional factors of maintenance self-efficacy, action planning, coping planning, and behaviors of hand washing and sleep hygiene. A further 2 months later, at Time 3, college students (*N* = 297) were asked to self-report on their hand washing and sleep hygiene behaviors over the past month. *Findings:* Data were analyzed using variance-based structural equation modelling. Results showed significant direct effects of attitudes, subjective norm, and perceived behavioral control on intentions; significant direct effects of action self-efficacy on maintenance self-efficacy; and significant direct effects of maintenance self-efficacy on action planning and coping planning. Significant direct effects of intention on action planning (sleep hygiene only), and significant direct effects of intention, maintenance self-efficacy (hand washing only), action and coping planning on behavior were also observed. Action planning also moderated the intention–behavior relationship, but only for hand washing. There were also significant total indirect effects of action self-efficacy on behavior mediated by maintenance self-efficacy, action planning, and coping planning for both behaviors, and significant total indirect effects of subjective norm and perceived behavioral control on behavior mediated by intention for sleep hygiene. When past behavior was included in the integrated model predicting all the psychological variables and behavior, all of the structural relations were attenuated. *Discussion:* Current findings indicate that college students’ hand washing and sleep hygiene behaviors are a function of both motivational and volitional factors. Findings also indicate that the TPB and HAPA pathways might differ for the two health behaviors. Implications of the current findings for future health interventions aimed at improving college students’ hand washing and sleep hygiene are discussed.

## 1. Introduction

Entering college is a key developmental stage and an important period for maintaining and cultivating health behaviors such as exercise, fruit and vegetable intake, hand washing, seatbelt use, and sleep hygiene; and for reducing health risk behaviors such as alcohol and drug use, smoking, and unprotected sex [1,2]. This is because the college years, as compared to the adolescent years, is a time where individuals experience more personal freedom and opportunities to develop their personal identities and cultivate new habits [3,4]. To understand and intervene to change health behavior, models and theories of social cognition have often been used. Such theories can help to identify the potentially modifiable factors that can be reliably related to behavior through various social psychological processes, and can be targeted in interventions designed to promote health behaviors [5,6]. In the current study, we aimed to identify the key social-cognition factors that underpinned two important health behaviors in college students: hand washing and sleep hygiene. In particular, we focused on identifying the motivational and volitional antecedents of college students’ hand washing and sleep hygiene behaviors based on an integrated model of behavior that combined social-cognition constructs from the Theory of Planned Behavior (TPB) [7,8] and Health Action Process Approach (HAPA) [9,10].

Hand washing with soap is an effective way to prevent the spread of communicable disease, such as respiratory and diarrheal illnesses [11,12]. However, hand washing compliance is poor, particularly in developing countries [13,14]. Previous research has mainly focused its attention on examining the social-cognition factors of hand hygiene among health care workers [15], with few studies targeting other population groups, such as college students [16]. In addition, social-cognition predictors of hand washing have been explored in the context of food hygiene [17,18,19] or the prevention of influenza infection [20], rather than on the hand washing behavior itself. Thus, it seems apparent that more formative, theory-based research is needed to inform the development of interventions to promote hand washing among college students [21,22].

In addition, also requiring attention is the poor sleep quality of college students [23,24]. Adequate and good quality sleep is important to the overall health, quality of life, and academic performance of college students [25,26]. Promoting good sleep hygiene practices, therefore, may be important to improving the sleep of college students. Although the influence of sleep hygiene on healthy sleep remains mixed [27], participating in good sleep hygiene could be argued to still be the most effective strategy for promoting sleep health, particularly on a population level [28]. Sleep hygiene refers to individuals following the recommended behaviors which promote the human body’s natural sleep-wake rhythms for restful sleep [29]. These behaviors include, for example, making the sleep environment such as the bedroom restful, avoiding stressful and anxiety-provoking activities before going to bed, and avoiding going to bed thirsty or hungry [30]. Although adopting sleep hygiene behaviors cannot guarantee good quality sleep, they are more likely to promote better sleep than if not followed [31]. It is therefore important to determine the theory-based social-cognition determinants of sleep hygiene among college students.

Social-cognition theories such as the TPB [7] and HAPA [9] have been used to explain and predict the health behaviors of both hand washing [16,32] and sleep hygiene [33,34]. According to the TPB, intention is viewed as the proximal and most important predictor of behavior, with intention proposed to be predicted by three belief-based constructs: attitudes, subjective norm, and perceived behavioral control. Attitude refers to the positive or negative evaluations towards the consequences of performing the intended behavior, subjective norm refers to perceived expectations of important others approving the intended behavior, and perceived behavioral control refers to perceptions of the ease or difficulty of performing the intended behavior [7]. Perceived behavioral control is also expected to have a direct effect on the intended behavior, to the extent that perceived behavioral control reflects actual control. The effects of TPB on predicting a range of health behaviors have been well-documented [35,36]. More specifically, and in the context of the current study, the TPB has been used to predict hand washing and sleep hygiene. For example, previous research has found support for the TPB constructs in predicting the intentions, and thus behavior, of caterers’ [17], health care workers’ [37,38], and nursing students’ [39] hand hygiene, and adolescents’ [40] and college students’ [41,42] sleep hygiene.

Although previous TPB research has consistently identified intentions as a determinant of health behaviors, the relationship between intentions and behavior is not perfect. Dual-phase models of behavior, such as the HAPA, propose self-regulatory strategies at the post-intentional volitional phase that can help individuals enact their intentions to implement the health behaviors [9,10]. For example, the self-regulatory strategy of planning is suggested to help an individual act on their intention, bridging the gap between intention and behavior [43]. According to the HAPA, there are two kinds of planning: action planning and coping planning. Action planning is a task-facilitating strategy (i.e., making plans of when, where, and how to perform the intended behavior) that connects the individual with good opportunities to act. Coping planning is a distraction-inhibiting strategy (i.e., making plans that anticipate challenging situations that might prevent an individual from performing the intended behavior) that protects good intentions from anticipated obstacles [9]. The HAPA also proposes perceived self-efficacy as an important self-regulatory strategy and is suggested to contribute to the intention formation, but is also proposed to be important at all stages of the health behavior change process [10]. In the HAPA, self-efficacy beliefs are considered to be phase-specific and, thus, several types of self-efficacy can be distinguished. Action self-efficacy is an optimistic belief during the pre-actional (motivational) phase, where an individual does not yet act, but is developing a motivation to do so through imagining success and anticipating potential outcomes of diverse strategies. Maintenance self-efficacy, on the other hand, is an optimistic belief during the post-actional (volitional) phase, where an individual perceives they have the competence and capability to persevere the formed action of behavior in the face of obstacles. A recent meta-analysis provided support for the HAPA constructs of planning and self-efficacy in predicting health behaviors [44], and previous research has also shown planning and self-efficacy to predict college students’ hand washing [45,46] and sleep hygiene [47] more specifically.

## 2. The Current Study and Hypotheses

**Figure 1:**
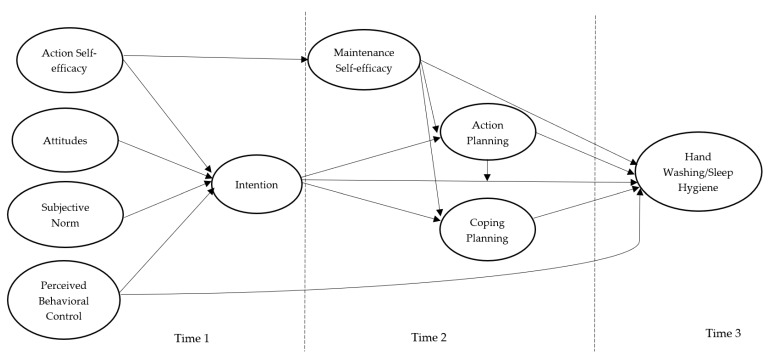
The proposed integrated model with social-cognition constructs from the Theory of Planned Behavior (TPB) and Health Action Process Approach (HAPA) for hand washing and sleep hygiene among college students. Note. All hypothesized effects among the psychological and behavioral variables were proposed to be positive in direction.

Given the importance of hand washing and sleep hygiene in college students, identifying the key theory-based determinants of these important health behaviors may provide important evidence on which to base interventions to promote participation in these behaviors. Moreover, limited studies on these health behaviors have been conducted in Eastern contexts such as China, and given cultural differences between Eastern and Western societies [48], generalizing findings from Western contexts to Eastern contexts may not be appropriate. Therefore, the purpose of the present study was to examine an integrated theoretical model of behavior that incorporated constructs from the TPB and HAPA to predict the hand washing and sleep hygiene behaviors of Chinese college students. Specifically, the TPB belief-based constructs of attitudes, subjective norm, perceived behavioral control, and intention were integrated with the HAPA constructs of action self-efficacy, maintenance self-efficacy, action planning, and coping planning. The approach rides on the cusp of recent research applying integrated theoretical models to explain behavior [6], and is one of the first to simultaneously account for two sets of processes (motivational and volitional) likely to impact on behavior (i.e., hand washing and sleep hygiene of Chinese college students) derived from theories of social cognition. The motivational pathways are represented by the effects of social-cognition constructs from the integration of the TPB (attitudes, subjective norm, perceived behavioral control, intention) and HAPA (action self-efficacy, intention). The volitional pathways are represented by the effects of social-cognition constructs from the HAPA (maintenance self-efficacy, action planning, coping planning). We outline the hypotheses in the next section. The hypotheses are illustrated in Figure 1.

.

Turning first to the motivational effects, it was expected that attitudes, subjective norm, perceived behavioral control, and action self-efficacy (Time 1) would predict intentions (Time 1); and intentions and perceived behavioral control (Time 1) would predict behavior (Time 3). It was also expected that action self-efficacy (Time 1) would predict maintenance self-efficacy (Time 2). Turning next to the volitional effects, it was expected that intention (Time 1) would predict action planning and coping planning (Time 2) and behavior (Time 3); maintenance self-efficacy (Time 2) would predict action planning and coping planning (Time 2); and maintenance self-efficacy, action planning, and coping planning (Time 2) would predict behavior (Time 3). It was further proposed that action planning would moderate the intention–behavior relationship consistent with the model of action phases [49]. As past behavior has shown to have pervasive effects on the key constructs of psychological theories such as TPB and HAPA, and substantially attenuating their relations with health behaviors [50,51], we further examined the model including past behavior as a predictor of all psychological variables and behavior. If it is demonstrated that past behavior attenuates all model effects to trivial values, then the model would be considered redundant [51]. It was expected that significant effects of past behavior on all constructs in the model would emerge, and that effects would be attenuated.

A set of indirect effects consistent with the premises of the TPB and HAPA were also specified. It was expected that attitudes, subjective norm, and perceived behavior control would predict action planning and coping planning via intention, and predict behavior via intention, action planning, and coping planning. It was also expected that action self-efficacy would predict action planning and coping planning via intention and maintenance self-efficacy, and predict behavior via intention, maintenance self-efficacy, action planning, and coping planning. Finally, it was expected that intention and maintenance self-efficacy would predict behavior via action and coping planning.

## 3. Method

### 3.1. Participants and Procedure

With the assistance of course instructors from a medical school in China, data collection was conducted during the second semester of the 2017–2018 academic year. A three-wave face-to-face data collection method was used with 1 month time intervals between surveys: baseline (Time 1), 1 month after baseline (Time 2), 1 month after Time 2 (Time 3). Participants were required to be college students aged 18–35 years. At Time 1 (*N* = 1106; 201 males, 904 females, and one unknown), college students completed a survey assessing the motivational variables of action self-efficacy, attitudes, subjective norm, perceived behavior control, and intention, and self-reported on their past hand washing and sleep hygiene behaviors over the previous month. Demographic factors of age, gender, and college majors were also measured. One month later, at Time 2, 524 (85 males and 439 females) of the college students completed a further survey measuring the volitional variables of maintenance self-efficacy, action planning, and coping planning, and again self-reported on their hand washing and sleep hygiene. One month after the Time 2 survey, at Time 3, 297 (52 males and 245 females) of the college students self-reported on their hand washing and sleep hygiene over the previous month. Data across each of the time points were matched using student numbers provided by the participants, and written informed consent was obtained. Attrition analyses using independent-samples T tests indicated that there were no significant differences in hand washing, sleep hygiene, and the motivational variables between participants that dropped out of the study and those who completed the Time 3 assessment. There were, however, differences in sleep hygiene (*t* (1009) = 2.424, *p* = 0.016) and hand washing intention (*t* (1091) = 2.102, *p* = 0.036). The study was conducted in accordance with the Declaration of Helsinki. The Research Ethics Committee from the Hong Kong Baptist University approved the study protocol (Ref No.: HASC17-18_0688).

### 3.2. Measures

*Hand washing*. Across all three time points, college students’ hand washing was measured by using the last month as the time-frame and asking whether they washed their hands at least 10 times each day as recommended by the WHO [52] in three situations relevant to college students: (a) before eating, cooking, and going to bed; (b) after going to the toilet, attending physical education class, coughing, sneezing, touching animals as well as going to public places; and (c) anytime they feel that their hands are dirty. College students were asked to rate on a four-point Likert scale ranging from (1) never to (4) always. A higher total score indicated good hand washing behavior.

*Sleep hygiene*. Across all three time points, college students’ sleep hygiene was measured by using the last month as the time-frame and asking whether they followed the seven good sleep hygiene behaviors recommended by the National Sleep Foundation of United States of America [53], including (a) limiting daytime naps to 30 min, (b) avoiding stimulants such as caffeine and nicotine close to bedtime, (c) exercising to promote good quality sleep, (d) steering clear of food that can be disruptive right before sleep, (e) ensuring adequate exposure to natural light, (f) establishing a regular relaxing bedtime routine, and (g) making sure that the sleep environment is pleasant. College students were asked to rate the degree to which they are able to follow the good practice by rating on a four-point Likert scale ranging from (1) never to (4) always. A higher total score indicated good sleep hygiene behavior.

*Intentions*. At Time 1, college students’ intentions of following the recommendations of the hand washing by WHO and sleep hygiene by National Sleep Foundation of United States of America were measured using three items. Sample items are “Following the recommendation of WHO on the good hand washing behaviors every day in the coming month is…something I intend to do” and “Following the recommendation of National Sleep Foundation of United States of America on the good sleep hygiene every day in the coming month is…something I plan to do”. Items were rated on a seven-point Likert scale, ranging from (1) strongly disagree to (7) strongly agree.

*Attitudes*. At Time 1, attitude was assessed using a common stem on hand washing “Following the recommendation of WHO on the good hand washing behaviors every day in the coming month for me is…” and sleep hygiene “Following the recommendation of National Sleep Foundation of United States of America on the good sleep hygiene every day in the coming month for me is…”, which is followed by six semantic differential items. Items were rated on a seven-point scale, ranging from one to seven: unpleasant–pleasant, meaningless–meaningful, bad–good, unhealthy–healthy, unenjoyable–enjoyable, and harmful–beneficial.

*Subjective norm*. At Time 1, subjective norm was assessed using five items measuring participants’ perceptions of their important others’ approval on the two behaviors of following the recommendations of the hand washing by WHO and sleep hygiene by National Sleep Foundation of United States of America. Sample items are “Following the recommendation of WHO on the good hand washing behaviors every day in the coming month is…something those most important person to me would allow me to do”; and “Following the recommendation of National Sleep Foundation of United States of America on the good sleep hygiene every day in the coming month is…something those most people expected me to do”. Items were rated on a seven-point Likert scale, ranging from (1) strongly disagree to (7) strongly agree.

*Perceived behavioral control*. At Time 1, perceived behavioral control was assessed using four items measuring participants’ perceptions of their control over following the recommendations of the hand washing by WHO and sleep hygiene by National Sleep Foundation of United States of America. Samples items are “Following the recommendation of WHO on the good hand washing behaviors every day in the coming month is…something I have full control”; and “Following the recommendation of National Sleep Foundation of United States of America on the good sleep hygiene every day in the coming month is…something easy for me to do”. Items were rated on a seven-point Likert scale, ranging from (1) strongly disagree to (7) strongly agree.

*Action self-efficacy*. At Time 1, action self-efficacy was assessed using three items measuring college students’ level of confidence on starting to act on the behaviors of following the recommendations of the hand washing by WHO and sleep hygiene by National Sleep Foundation of United States of America. Samples items are “If you have not followed the recommendation of WHO on the good hand washing yet, do you have the confidence to start to follow the recommendation…even if you have to force myself doing so at the current stage”; and “If you have not followed the recommendation of National Sleep Foundation of United States of America on the good sleep hygiene yet, do you have the confidence to start to follow the recommendation…even if it consumes a lot of energy”. Items were rated on a four-point Likert scale, ranging from (1) totally disagree to (4) totally agree.

*Maintenance self-efficacy*. At Time 2, maintenance self-efficacy was assessed using three items measuring college students’ level of confidence of maintaining the behaviors of following the recommendations of the hand washing by WHO and sleep hygiene by National Sleep Foundation of United States of America in the long term. Samples items are “If you are able to follow the recommendation of WHO on the good hand washing last month, do you have the confidence to maintain it in the long term…even if you cannot immediately experience the benefits of maintain a good sleep hygiene”; and “If you are able to follow the recommendation of National Sleep Foundation of United States of America on the good sleep hygiene last month, do you have the confidence to maintain it in the long term…even if it may take a long time to turn it into a habit of yourself”. Items were rated on a four-point Likert scale, ranging from (1) totally disagree to (4) totally agree.

*Action planning*. At Time 2, action planning was measured by three items assessing the extent to which participants had made a plan, in terms of how, when, and with whom to perform the behaviors of following the recommendations of the hand washing by WHO and sleep hygiene by National Sleep Foundation of United States of America in the coming month [54]. Sample items are “In the coming month I have made a detailed plan regarding…how to maintain the recommendations of good sleep hygiene”; and “In the coming month I have made a detailed plan regarding…when to maintain the recommendations of hand washing behavior”. Items were rated on a four-point Likert scale, ranging from (1) totally disagree to (4) totally agree.

*Coping planning*. At Time 2, coping planning was measured by three items assessing the extent to which participants had made a plan to cope with challenging circumstances that may arise regarding the maintenance of the behaviors of following the recommendations of the hand washing by WHO and sleep hygiene by National Sleep Foundation of United States of America in the long term. Sample items are “In the coming month I have made a coping plan of how to maintain the recommendations of good sleep hygiene under disadvantaged circumstances… facing the unexpected situation like nigh party invitation from friends”; and “In the coming month I have made a coping plan of how to maintain the recommendations of hand washing under disadvantaged circumstances…facing the uncontrolled situation like no soap or clean water”. Items were rated on a four-point Likert scale, ranging from (1) totally disagree to (4) totally agree.

*Demographic variables*. College students self-reported their gender, age in years, and college majors undertaken.

### 3.3. Data Analysis

**Figure 2:**
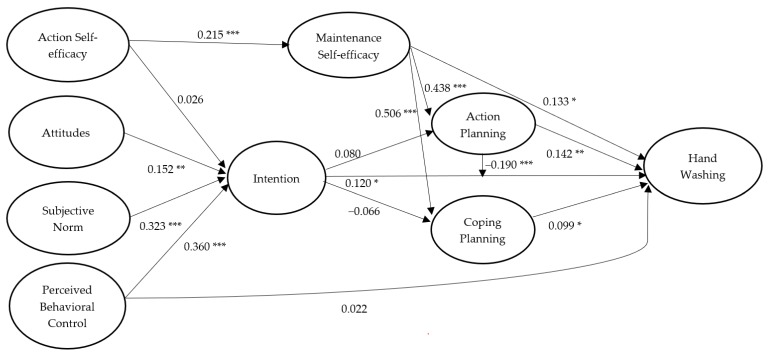
Standardized path coefficients of the Integrated Social-Cognition Model for hand washing. Note. College students’ age and gender were controlled. All hypothesized effects among the psychological and behavioral variables were proposed to be positive in direction. * *p* < 0.05; ** *p* < 0.01; *** *p* < 0.001.

**Figure 3:**
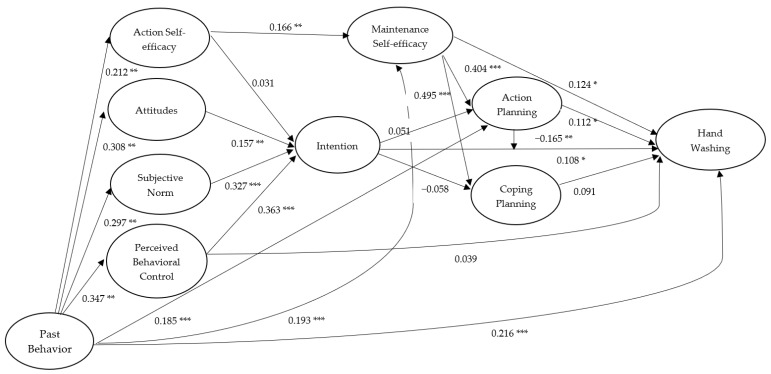
Standardized path coefficients of the Integrated Social-Cognition Model for hand washing including past behavior. Note. College students’ age and gender were controlled. All hypothesized effects among the psychological and behavioral variables were proposed to be positive in direction. Only significant effects from past behavior to psychological variables were displayed. * *p* < 0.05; ** *p* < 0.01; *** *p* < 0.001.

**Figure 4:**
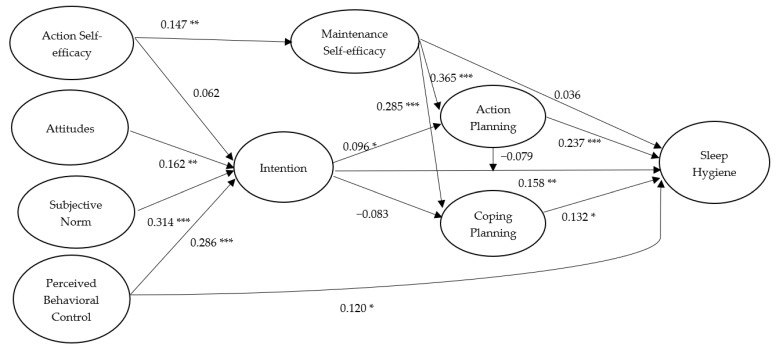
Standardized path coefficients of the Integrated Social-Cognition Model for sleep hygiene. Note. College students’ age and gender were controlled. All hypothesized effects among the psychological and behavioral variables were proposed to be positive in direction. * *p* < 0.05; ** *p* < 0.01; *** *p* < 0.001.

**Figure 5:**
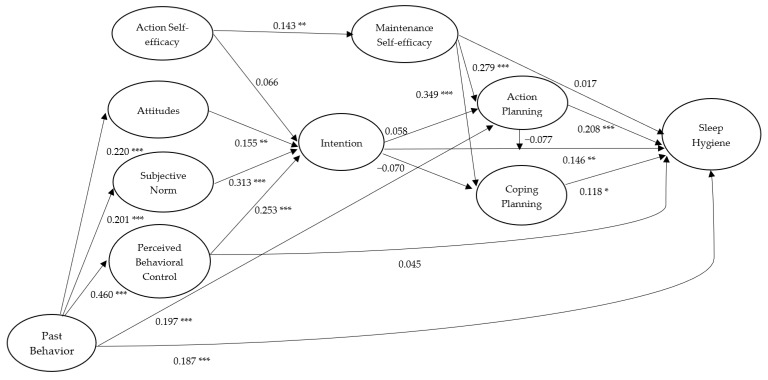
Standardized path coefficients of the Integrated Social-Cognition Model for sleep hygiene including past behavior. Note. College students’ age and gender were controlled. All hypothesized effects among the psychological and behavioral variables were proposed to be positive in direction. Only significant effects from past behavior to psychological variables were displayed. * *p* < 0.05; ** *p* < 0.01; *** *p* < 0.001.

We conducted a preliminary analysis on the descriptive data using the software of SPSS Statistics 25 (IBM Corp., 2017, Armonk, NY, USA). We tested the proposed integrative model for both the health behaviors of hand washing and sleep hygiene (see Figure 1) using the variance-based structural equation modeling (VB-SEM) within the Warp PLS v6.0 software (ScriptWarp Systems, Laredo, TX, USA) [55]. We used VB-SEM as it is has more statistical power, as VB-SEM is based on ranked data and, thus, less affected by issues such as model complexity and non-normality. The VB-SEM approach is similar to the traditional covariance-based structural equation modeling in that latent factors were used to explicitly model the measurement errors. As such, it is optimal to use VB-SEM to estimate the proposed integrated model of hand washing and sleep hygiene in the current study. We set items for demographic, psychological, and behavioral variables as the indicators of formative latent variables, while all paths were set as free parameters in VB-SEM. Furthermore, we tested alternative models with past behavior (i.e., hand washing and sleep hygiene measured at Time 1) predicting all psychological and behavioral variables. The paths from demographic variables to each of the psychological and behavioral variables were controlled, although they were not displayed in Figure 1, Figure 2, Figure 3, Figure 4 and Figure 5.

To ensure the validity of the measurement model, reliability and validity indicators of each measure were also tested. Firstly, the factor loadings of items on its indictors and the composite reliability coefficients (ρ) of each measure are expected to be over 0.700. In addition, the average variance extracted (AVE) is expected to be over 0.500, indicating sufficient variance of the underlying factor was accounted for by its items. Multiple criteria were used to evaluate the overall goodness of fit of the proposed models. The goodness-of-fit (GoF) index can be viewed as small, medium, and large effect sizes, with values of 0.100, 0.250, and 0.360, respectively [56]. It is also expected that, for an adequate model, the average path coefficient (APC) and the average R^2^ (ARS) are significantly different from zero. Furthermore, it is expected that, for a well-fitting model, the values of average variance inflation factor (AVIF) for model parameters should be less than 5.000 [55].

## 4. Results

### 4.1. Preliminary Analyses

The means, standard deviations, composite reliabilities, and correlations for hand washing and sleep hygiene are presented in Table 1 and Table 2, respectively. For all measures, reliability coefficients were above the cut-off criteria of 0.70, except baseline sleep hygiene (ρ = 0.677). For hand washing, correlations among psychological variables and behavior variables were generally positive and significant. However, the correlations between attitudes, action self-efficacy, intention, and coping planning were nonsignificant. Moreover, the correlation between action self-efficacy and hand washing at Time 3 was nonsignificant. For sleep hygiene, correlations among psychological variables and behavior variables were also generally positive and significant. However, no significant correlations were found between coping planning and attitudes, subjective norm, action self-efficacy, intention, and sleep hygiene at Time 3, while no significant relations were found between attitudes, subjective norm, and action planning. In addition, no significant associations were found between attitudes, action self-efficacy, maintenance self-efficacy, and sleep hygiene at Time 1. The associations between attitudes and maintenance self-efficacy as well as between subjective norm and action self-efficacy were not significant. For hand washing and sleep hygiene, we presented model variables’ composite reliabilities and intercorrelations in Table 1 and Table 2, respectively.

The construct validities of the measurement for both hand washing and sleep hygiene were confirmed in the VB-SEM. According to the multiple model fit and quality indices, the overall fit of the proposed model of hand washing excluding past behavior is acceptable (GoF Index = 0.307; APC = 0.126, *p* = 0.007, ARS = 0.143, *p* = 0.003; AVIF = 1.135). Including past behavior of hand washing into the proposed model improves the multiple model fit and quality indices (GoF Index = 0.356; APC = 0.135, *p* = 0.004, ARS = 0.197, *p* < 0.001; AVIF = 1.134). Likewise, the overall fit of the proposed model of sleep hygiene excluding past behavior is acceptable (GoF Index = 0.249; APC = 0.122, *p* = 0.008, ARS = 0.105, *p* = 0.017; AVIF = 1.070). Including past behavior of sleep hygiene into the proposed model improves the multiple model fit and quality indices (GoF Index = 0.289; APC = 0.122, *p* = 0.008, ARS = 0.105, *p* = 0.017; AVIF = 1.070). The values of AVE for most of the measured variables were above 0.500, except the subjective norm and past behavior of sleep hygiene.

### 4.2. Model Effects

For hand washing, standardized parameter estimates for the hypothesized structural relations of the integrated model with past behavior excluded are presented in Figure 2. Regarding the paths based on the TPB model, it was revealed that attitudes (*β* = 0.152, *p* = 0.004), subjective norm (*β* = 0.323, *p* < 0.001), and perceived behavioral control (*β* = 0.360, *p* < 0.001) significantly predicted intentions of hand washing, and intentions predicted hand washing behavior at Time 3 (*β* = 0.120, *p* = 0.018). The direct effect of perceived behavioral control on hand washing at Time 3 was not significant (*β* = 0.022, *p* = 0.351). Although the effect of action self-efficacy on intention was nonsignificant (*β* = 0.026, *p* = 0.324), the effect of action self-efficacy on maintenance self-efficacy was significant (*β* = 0.215, *p* < 0.001), and maintenance self-efficacy significantly predicted hand washing at Time 3 (*β* = 0.133, *p* = 0.010). Maintenance self-efficacy also significantly predicted action planning (*β* = 0.438, *p* < 0.001) and coping planning (*β* = 0.506, *p* < 0.001), and action planning (*β* = 0.142, *p* = 0.007) and coping planning (*β* = 0.099, *p* = 0.043) predicted hand washing at Time 3. Effects of intention on action planning (*β* = 0.080, *p* = 0.083) and coping planning (*β* = −0.066, *p* = 0.126) were not significant. Moreover, the moderation effect of action planning on the intention-hand washing behavior relationship was significant (*β* = −0.190, *p* < 0.001). In terms of the indirect effects, action self-efficacy significantly predicted action planning (*β* = 0.096, *p* = 0.047) and coping planning (*β* = 0.107, *p* = 0.031) via maintenance self-efficacy. Furthermore, maintenance self-efficacy predicted hand washing at Time 3 (*β* = 0.112, *p* = 0.025) via the action planning and coping planning. We present a full breakdown of estimates on the integrated model of hand washing, including direct, indirect, and total effects in the table in Table S1, which also includes the effects from the control variables (age and gender).

When past behavior (i.e., hand washing at Time 1) was included in the integrated model predicting all the psychological variables and hand washing at Time 3, all of the structural relations were attenuated (see Figure 3). Specifically, the effect of coping planning on hand washing at Time 3 became nonsignificant (*β* = 0.091, *p* = 0.057). Past behavior had significant effects on all the psychological variables and hand washing at Time 3 but not on intentions (*β* = 0.027, *p* = 0.322) and coping planning (*β* = 0.066, *p* = 0.124). In terms of the indirect effects, past behavior had an effect on intentions via action self-efficacy, attitudes, subjective norm, and perceived control (*β* = 0.278, *p* < 0.001). Further, the sum of indirect effects of past behavior on action planning (*β* = 0.108, *p* = 0.030) and coping planning (*β* = 0.095, *p* = 0.049) via action self-efficacy, attitudes, subjective norm, perceived behavior control, intention, and maintenance self-efficacy were significant. Additionally, the sum of indirect effect of past behavior on hand washing at Time 3 via all the psychological variables was significant (*β* = 0.122, *p* = 0.016). Direct effects from past behavior and control variables (age and gender) to all the psychological variables and hand washing at Time 3 as well as the significant indirect effects are available in a separate table in Table S2.

For sleep hygiene, standardized parameter estimates for the hypothesized structural relations of the integrated model with past behavior excluded are presented in Figure 4. For the paths based on the TPB model, it was revealed that attitudes (*β* = 0.162, *p* = 0.002), subjective norm (*β* = 0.314, *p* < 0.001), and perceived behavioral control (*β* = 0.286, *p* < 0.001) significantly predicted intentions of sleep hygiene, and intentions predicted sleep hygiene at Time 3 (*β* = 0.158, *p* = 0.003). A significant direct effect of perceived behavioral control on sleep hygiene at Time 3 was also found (*β* = 0.120, *p* = 0.018). Although the effect of action self-efficacy on intentions was nonsignificant (*β* = 0.062, *p* = 0.141), a significant effect of action self-efficacy on maintenance self-efficacy was found (*β* = 0.147, *p* = 0.005). Further, although the direct effect of maintenance self-efficacy on sleep hygiene at Time 3 was nonsignificant (*β* = 0.036, *p* = 0.267), the effects of maintenance self-efficacy on action planning (*β* = 0.361, *p* < 0.001) and coping planning (*β* = 0.285, *p* < 0.001) were significant, and action planning (*β* = 0.237, *p* < 0.001) and coping planning (*β* = 0.132, *p* = 0.010) also predicted sleep hygiene at Time 3. It should be noted that intention also significantly predicted action planning (*β* = 0.096, *p* = 0.047) but not coping planning (*β* = −0.083, *p* = 0.075). Moreover, the moderation effect of action planning on the intention–sleep hygiene relationship was nonsignificant (*β* = −0.079, *p* = 0.084). In terms of the indirect effects, we only found that maintenance self-efficacy significantly predicted sleep hygiene at Time 3 via action planning and coping planning (*β* = 0.123, *p* = 0.016). The full breakdown of estimates on the integrated model of sleep hygiene including direct, indirect, and total effects are presented in the table in Table S3, including the effects of control variables: age and gender.

When past behavior (i.e., sleep hygiene at Time 1) was included in the integrated model predicting all the psychological variables and sleep hygiene at Time 3, all of the structural relations were attenuated (see Figure 5). Past behavior significantly predicted attitudes (*β* = 0.220, *p* < 0.001), subjective norm (*β* = 0.201, *p* < 0.001), perceived behavioral control (*β* = 0.460, *p* < 0.001), action planning (*β* = 0.197, *p* < 0.001), and sleep hygiene at Time 3 (*β* = 0.187, *p* = 0.088), but not the other psychological variables of action self-efficacy (*β* = 0.085, *p* = 0.069), intention (*β* = 0.078, *p* = 0.088), maintenance self-efficacy (*β* = 0.070, *p* = 0.110), and coping planning (*β* = 0.085, *p* = 0.069). In terms of the indirect effects, past behavior significantly predicted intentions of sleep hygiene via action self-efficacy, attitudes, subjective norm, and perceived behavioral control (*β* = 0.219, *p* < 0.001). In addition, past behavior had a significant indirect effect on future behavior, that is, sleep hygiene at Time 3, via the psychological variables (*β* = 0.126, *p* = 0.014). Direct effects from past behavior and control variables (age and gender) in the integrated model of sleep hygiene are available in a separate table in Table S4. 

## 5. Discussion

The purpose of the present study was to identify the antecedents of Chinese college students’ hand washing and sleep hygiene behaviors based on an integrated model of behavior that combined social-cognition constructs from the TPB and HAPA. Results indicated significant effects of motivational and volitional factors on college students’ hand washing and sleep hygiene. Specifically, results showed significant direct effects of attitudes, subjective norm, and perceived behavioral control on intentions; significant direct effects of action self-efficacy on maintenance self-efficacy; and significant direct effects of maintenance self-efficacy on action planning and coping planning for both behaviors. Results also revealed significant direct effects of intention on action planning (sleep hygiene only), and significant direct effects of intention, maintenance self-efficacy (hand washing only), action and coping planning on behavior were also observed. Action planning moderated the intention–behavior relationship, but only for hand washing. There were also significant total indirect effects of action self-efficacy on behavior mediated by maintenance self-efficacy, action planning, and coping planning for both behaviors, and significant total indirect effects of subjective norm and perceived behavioral control on behavior mediated by intention for sleep hygiene. When past behavior was included in the integrated model predicting all the psychological variables and behavior, effect sizes of structural relations were attenuated. Overall, findings of the current study confirmed the TPB pathways of attitudes, subjective norm, and perceived behavioral control on intentions and hand washing and sleep hygiene behaviors; as well as the HAPA pathways of action self-efficacy to hand washing and sleep hygiene behaviors via the maintenance self-efficacy, action and coping planning. Findings of the current study may inform the development of interventions building on integrated social-cognition models to promote hand washing and sleep hygiene among college students.

Regarding the motivational phase of the proposed integrated model, findings of the current study on both hand washing and sleep hygiene were confirmed and are in line with previous meta-analyses on the application of TPB predicting health behaviors [35,36]. Specifically, the TPB constructs of attitudes, subjective norm, and perceived behavioral control significantly predicted hand washing and sleep hygiene intentions, while intentions further predicted hand washing and sleep hygiene behaviors. This is also in line with the findings of previous studies using the TPB to predict individuals’ hand washing [37,38,39] and sleep hygiene [40,41,42]. It is also important to note that perceived behavioral control had a direct effect on sleep hygiene, consistent with TPB predictions [35]. This indicates that when college students’ perceived control on sleep hygiene reflects their actual control, it is a direct determinant of their behavior independent of intentions. However, surprisingly, we found that the effect from action self-efficacy to intention was nonsignificant. Findings suggest that beliefs in personal capacity to perform the behavior, captured by action self-efficacy, may be less important for these behaviors than beliefs relating to barriers and facilitators of the behavior, that tend to be captured by perceived behavioral control. Future health promotion programs aimed at promoting hand washing and sleep hygiene among college students should consider including targeting change in three sets of beliefs: (a) behavioral beliefs regarding the anticipated outcomes of performing the behaviors of hand washing and sleep hygiene; (b) normative beliefs about college students’ perception of the views of others on performing the behaviors, in particular important others; and (c) control beliefs that related to perceived capability to perform the behaviors [8,57].

Testing the volitional stage is informed by the HAPA theoretical framework [9,44]. In the current study, the indirect effect of action self-efficacy on hand washing and sleep hygiene behaviors via maintenance self-efficacy, action planning, and coping planning was confirmed. That is, college students’ action self-efficacy predicted maintenance self-efficacy, which further predicted hand washing and sleep hygiene via action planning and coping planning. This indicates that the mechanism by which college students’ confidence beliefs relate to hand washing and sleep hygiene behaviors is independent of intentions, but, instead, directed though beliefs regarding maintaining the behaviors and plans to perform the behavior [34]. With regard to hand washing, maintenance self-efficacy was also directly related to behavior. This effect might indicate that college students’ confidence beliefs in preserving their hand washing behavior in the face of obstacles can directly lead to the performance of the behavior. Moreover, the negative moderating effect of action planning on the intention–behavior relation indicates that college students with high levels of action planning will be less likely to enact their intentions to perform hand washing. Although previous research has identified that action planning moderates the intention–behavior relationship, the effects are typically positive [58,59]. To speculate, one reason for the negative moderating effects may be that individuals who form action plans tend not to rely on intentions because, most likely, they have probably acted out of habit, which is non-intentional [60]. For sleep hygiene, intention predicted action planning but not coping planning. This might be due to the fact that sleep is routine behavior for college students as intention can directly lead to sleep hygiene without having to make coping plans to manage anticipated challenging situations. Further, maintenance self-efficacy of sleep hygiene was not directly related to the behavior but was indirectly related to sleep hygiene via the action and coping planning. This indicates that college students’ beliefs in their confidence to maintain good sleep hygiene might have to go through the planning process in order to facilitate performance of the behavior. In future, interventions aimed at promoting the maintenance of hand washing and sleep hygiene behaviors should target change in maintenance self-efficacy and action and coping planning. In other words, the promotion of perceived confidence on initiating and maintaining the behaviors should be paired up with promoting capacity to make plans.

A key contribution of the current research is the confirmation of multiple pathways by which college students’ psychological constructs affect their hand washing and sleep hygiene behaviors. Consistent with models of social cognition, the constructs from the motivational phase in the integrated model predicted these two important health behaviors. However, we also found that constructs underpinning volitional processes accounted for significant variance in behavior, independent of the motivational constructs. Specifically, it seems that phase-specific types of self-efficacy (i.e., maintenance self-efficacy) and planning are important unique predictors of behavior. It is interesting, however, that for both behaviors current findings failed to demonstrate a relationship between action self-efficacy and intention, and also a relationship between intention and coping planning. A number of potential interpretations of these patterns of effects exist. It is possible, for example, that both hand washing and sleep hygiene might be considered simple health behaviors which are less likely to rely on perceptions of competence to initiate the behavior compared to other complex behaviors such as physical activity [60,61]. Further, coping planning did not account for the intention-behavior effect, as predicted by the HAPA [9], but predicted behavior directly. This suggests that different processes may have a stronger impact on behavior, and that the behavior of some participants may be controlled more by intentional factors while others may be controlled more by volitional factors. Future research will need to explore this distinction further, identifying what moderators will determine whether the intentional and volitional processes predominate in determining students’ hand washing and sleep hygiene behaviors.

A further contribution of the current study is related to the findings for past behavior on hand washing and sleep hygiene. In the current study, the size of the model effects, especially effects of model constructs on future behavior, were attenuated, but most effects still hold after the inclusion of past behavior. Findings are in line with results of meta-analysis on the role of past behavior on key model constructs [35,51], in which past behavior is viewed as a predominant predictor that reflects the stability of the behavior itself [60,62]. This is because past behavior can reflect the non-conscious automatic processes leading to the behaviors, consistent with habitual effects [63,64]. Furthermore, control for the effects of past behavior in the integrated model is necessary to confirm the sufficiency of the model in predicting hand washing and sleep hygiene behaviors. Future research should explore the role of past behavior as a determinant of hand washing and sleep hygiene among college students, particularly the extent to which residual effects of past behavior may be accounted for by habit [65].

## 6. Strengths and Limitations 

The use of integrated theoretical approaches to examine effects of psychological constructs that represent multiple processes on behavior in a single testable model is emerging [6]. Adoption of such model is a major strength of the current study. The findings from this study add to the expanding evidence by identifying the motivational and volitional antecedents of college students’ hand washing and sleep hygiene behaviors based on an integrated model of behavior that combines social-cognitive constructs from the TPB and HAPA. A further strength was the use of a longitudinal design, many studies testing these theoretical approaches use cross-sectional designs. Finally, two health behaviors were examined, showing similar patterns of prediction for the integrated model, therefore providing confirmation of model effects.

Limitations of the current study should also be acknowledged. Despite the use of a longitudinal design, we recognize that our results do not permit the inference of causality. It must be stressed that any causal inferences are based on theory, not the data. Future studies should consider applying the experimental or randomized controlled intervention designs to test effects of manipulating model constructs on other constructs in the model, and on participation in the two health behaviors. Such designs will also allow a test of the mechanisms of action of the intervention through the theoretical constructs identified in the model [66,67]. Second, we relied on self-reported measures of behavior which might be affected by participants’ ability to recall their actions. Future studies might consider applying an objective means to measure the behaviors or using ecological momentary assessment [68]. Third, we measured hand washing and sleep hygiene in terms of the degree to which college students perform these good practices rather than the actual frequency of health behaviors (e.g., how many times you washed your hands per day) and behavioral outcomes (e.g., the quality and quantity of sleep). Future studies might also consider measuring the long-term effects of performing these behaviors on physical and mental health outcomes. Fourth, the attrition rate from baseline to the last wave of data collection was quite high. Although it is expected in longitudinal research, future studies should be mindful of adopting strategies to prevent the loss of participants to follow-up. Fifth, we directly assessed the behavioral, normative, and control beliefs of hand washing and sleep hygiene without conducting elicitation studies in advance. Elicitation studies are highly recommended when using the TPB to establish the salient beliefs that underpin their intentions to perform health behaviors [69]. Sixth, we did not fully comply with Ajzen’s correspondence rule when defining hand washing and sleep hygiene behaviors. This means that measure of the model constructs and behavior should correspond in terms of target, action, context, and time frame (TACT) [70]. Future studies should pay due diligence to measurement correspondence consistent with TACT principles. For example, in the current study, a better definition of sleep hygiene might be to refer to sleep hygiene as something people intend to do each time they prepare to go to sleep. Finally, data was collected from one medical school in China. Although the sample size was sufficiently large, future studies should consider collecting data from a more representative sample such as college students from different colleges and in different regions of China.

## 7. Conclusions

The current study tested an integrated model of behavior that combined social cognition constructs from TPB and HAPA to identify the motivational and volitional antecedents of hand washing and sleep hygiene among Chinese college students. Consistent with TPB predictions, attitudes, subjective norm, and perceived behavioral control predicted intentions, and intentions predicted hand washing and sleep hygiene behaviors. In addition, stage-specific self-efficacy and planning were also important predictors in the model. Consistent with the HAPA, action self-efficacy predicted maintenance self-efficacy, while maintenance self-efficacy predicted hand washing and sleep hygiene behaviors through the mediation of action planning and coping planning. The inclusion of past behavior attenuated the effects sizes of all predictors in the model for both hand washing and sleep hygiene behavior. These findings indicate the importance of accounting for past behavior and the need to identify possible mediators of its residual effects on future behavior, such as measures of habit. Overall, findings of the current study indicate that future interventions for improving hand washing and sleep hygiene among college students should target the key social-cognition factors found to predict behavior in the current study: behavioral, normative, and control beliefs, intentions, action and maintenance self-efficacy, and action and coping planning.

## Figures and Tables

**Table 1:** Means, standard deviations (SDs), reliabilities, and factor intercorrelations of the Integrated Model for Hand Washing.

Variables	Mean	SD	1	2	3	4	5	6	7	8	9	10
1. Attitudes T1	6.07	0.95	0.901									
2. Subjective norm T1	5.39	0.93	0.334 **	0.884								
3. PBC T1	5.55	0.89	0.353 **	0.476 **	0.861							
4. Action SE T1	3.00	0.77	0.144 *	0.205 **	0.250 **	0.873						
5. Intention T1	5.76	0.84	0.373 **	0.544 **	0.542 **	0.212 **	0.841					
6. Hand washing T1	3.40	0.50	0.331 **	0.292 **	0.367 **	0.196 **	0.216 **	0.743				
7. Maintenance SE T2	2.90	0.53	0.121 *	0.129 *	0.173 **	0.175 **	0.204 **	0.128 *	0.792			
8. Action planning T2	2.85	0.53	0.123 *	0.159 **	0.211 **	0.142 *	0.126 *	0.208 **	0.444 **	0.800		
9. Coping planning T2	2.80	0.54	0.111	0.124 *	0.133 *	0.053	0.065	0.156 **	0.507 **	0.522 **	0.767	
10. Hand washing T3	3.30	0.58	0.216 **	0.165 **	0.159 **	0.071	0.204 **	0.312 **	0.280 **	0.272 **	0.272 **	0.826

Note. PBC = perceived behavioral control; SE = self-efficacy; composite reliability is displayed on the diagonal line. T1 = Time 1; T2 = Time 2; T3 = Time 3; * *p* < 0.05; ** *p* < 0.01.

**Table 2:** Means, standard deviations (SDs), reliabilities, and factor intercorrelations of the Integrated Model for Sleep Hygiene.

Variables	Mean	SD	1	2	3	4	5	6	7	8	9	10
1. Attitudes T1	5.19	0.90	0.812									
2. Subjective norm T1	5.26	0.84	0.145 *	0.807								
3. PBC T1	4.91	10.01	0.158 *	0.381 **	0.854							
4. Action SE T1	2.67	0.65	0.141 *	0.113	0.254 **	0.810						
5. Intention T1	5.44	0.92	0.205 **	0.441 **	0.464 **	0.220 **	0.864					
6. Sleep hygiene T1	3.05	0.42	0.006	0.193 **	0.445 **	0.065	0.254 **	0.677				
7. Maintenance SE T2	2.83	0.53	0.043	0.196 **	0.252 **	0.135 *	0.232 **	0.072	0.807			
8. Action planning T2	2.74	0.51	0.085	0.081	0.237 **	0.182 **	0.124 *	0.222 **	0.336 **	0.763		
9. Coping planning T2	2.60	0.57	−0.010	0.014	0.146*	0.070	0.042	0.134 *	0.282 **	0.249 **	0.762	
10. Sleep hygiene T3	3.06	0.44	0.181 **	0.201 **	0.270 **	0.092	0.255 **	0.327 **	0.151*	0.267 **	0.071	0.746

Note. PBC = perceived behavioral control; SE = self-efficacy; composite reliability is displayed on the diagonal line. T1 = Time 1; T2 = Time 2; T3 = Time 3; * *p* < 0.05; ** *p* < 0.01.

## Supplementary Materials

The following are available online at https://www.mdpi.com/1660-4601/17/4/1209/s1, Table S1: Standardized Parameter Estimates for the Direct, Indirect, and Total Effects in the Integrated Model of Hand Washing Excluding Past Behavior, Table S2: Standardized Parameter Estimates for the Direct, Indirect, and Total Effects in the Integrated Model of Hand Washing Including Past Behavior, Table S3: Standardized Parameter Estimates for the Direct, Indirect, and Total Effects in the Integrated Model of Sleep Hygiene Excluding Past Behavior, Table S4: Standardized Parameter Estimates for the Direct, Indirect, and Total Effects in the Integrated Model of Sleep Hygiene Including Past Behavior.

## References

[1] Raynor D.A., Levine H. (2009). Associations between the five-factor model of personality and health behaviors among college students. J. Am. Coll. Health.

[2] Scott-Sheldon L.A.J., Carey K.B., Carey M.P. (2007). Health behavior and college students: Does Greek affiliation matter?. J. Behav. Med..

[3] Gardner B., de Bruijn G.-J., Lally P. (2012). Habit, identity, and repetitive action: A prospective study of binge drinking in UK students. Br. J. Health Psychol..

[4] Verplanken B., Sui J. (2019). Habit and identity: Behavioral, cognitive, affective, and motivational facets of an integrated self. Front. Psychol..

[5] Armitage C.J., Conner M. (2000). Social cognition models and health behaviour: A structured review. Psychol. Health.

[6] Hagger M.S., Hamilton K., Hagger M.S., Cameron L., Hamilton K., Hankonen N., Lintunen T. (2020). Changing behaviour using integrated theories. The Handbook of Behavior Change.

[7] Ajzen I. (1991). The theory of planned behavior. Organ. Behav. Hum. Decis. Process..

[8] Ajzen I., Schmidt P., Hagger M.S., Cameron L., Hamilton K., Hankonen N., Lintunen T. (2020). Changing behaviour using the theory of planned behavior. The Handbook of Behavior Change.

[9] Schwarzer R. (2008). Modeling health behavior change: How to predict and modify the adoption and maintenance of health behaviors. Appl. Psychol..

[10] Schwarzer R., Hamilton K., Hagger M.S., Cameron L., Hamilton K., Hankonen N., Lintunen T. (2020). Changing behaviour using the health action process approach. The Handbook of Behavior Change.

[11] Rabie T., Curtis V. (2006). Handwashing and risk of respiratory infections: A quantitative systematic review. Trop. Med. Inter. Health.

[12] Freeman M.C., Stocks M.E., Cumming O., Jeandron A., Higgins J.P., Wolf J., Prüss-Ustün A., Bonjour S., Hunter P.R., Fewtrell L. (2014). Hygiene and health: Systematic review of handwashing practices worldwide and update of health effects. Trop. Med. Inter. Health.

[13] Curtis V., Cairncross S. (2003). Effect of washing hands with soap on diarrhoea risk in the community: A systematic review. Lancet Infect. Dis..

[14] Von Lengerke T., Lutze B., Krauth C., Lange K., Stahmeyer J.T., Chaberny I.F. (2017). Promoting hand hygiene compliance: PSYGIENE—A cluster-randomized controlled trial of tailored interventions. Dtsch. Arztebl. Int..

[15] Korniewicz D.M., El-Masri M. (2010). Exploring the factors associated with hand hygiene compliance of nurses during routine clinical practice. Appl. Nurs. Res..

[16] Lhakhang P., Lippke S., Knoll N., Schwarzer R. (2015). Evaluating brief motivational and self-regulatory hand hygiene interventions: A cross-over longitudinal design. BMC Public Health.

[17] Clayton D.A., Griffith C.J. (2008). Efficacy of an extended theory of planned behaviour model for predicting caterers’ hand hygiene practices. Int. J. Environ. Health Res..

[18] Mullan B.A., Wong C.L. (2009). Hygienic food handling behaviours. An application of the Theory of Planned Behaviour. Appetite.

[19] Soon J.M., Baines R., Seaman P. (2012). Meta-analysis of food safety training on hand hygiene knowledge and attitudes among food handlers. J. Food Prot..

[20] Miller S., Yardley L., Little P. (2012). Development of an intervention to reduce transmission of respiratory infections and pandemic flu: Measuring and predicting hand-washing intentions. Psychol. Health Med..

[21] Mariwah S., Hampshire K., Kasim A. (2012). The impact of gender and physical environment on the handwashing behaviour of university students in Ghana. Trop. Med. Inter. Health.

[22] Mead M.P., Irish L.A. (2019). Application of health behaviour theory to sleep health improvement. J. Sleep Res..

[23] Thumma J., Aiello A.E., Foxman B. (2009). The association between handwashing practices and illness symptoms among college students living in a university dormitory. Am. J. Infect. Control..

[24] Buboltz Jr W., Jenkins S.M., Soper B., Woller K., Johnson P., Faes T. (2009). Sleep habits and patterns of college students: An expanded study. J. Coll. Counsel..

[25] Tsui Y.Y., Wing Y.K. (2009). A study on the sleep patterns and problems of university business students in Hong Kong. J. Am. Coll. Health.

[26] Becker C.M., Adams T., Orr C., Quilter L. (2008). Correlates of quality sleep and academic performance. Health Educ..

[27] Stepanski E.J., Wyatt J.K. (2003). Use of sleep hygiene in the treatment of insomnia. Sleep Med. Rev..

[28] Irish L.A., Kline C.E., Gunn H.E., Buysse D.J., Hall M.H. (2015). The role of sleep hygiene in promoting public health: A review of empirical evidence. Sleep Med. Rev..

[29] Pilcher J.J., Ginter D.R., Sadowsky B. (1997). Sleep quality versus sleep quantity: Relationships between sleep and measures of health, well-being and sleepiness in college students. J. Psychosom. Res..

[30] Mastin D.F., Bryson J., Corwyn R. (2006). Assessment of sleep hygiene using the Sleep Hygiene Index. J. Behav. Med..

[31] Kor K., Mullan B.A. (2011). Sleep hygiene behaviours: An application of the theory of planned behaviour and the investigation of perceived autonomy support, past behaviour and response inhibition. Psychol. Health.

[32] Todd J., Mullan B. (2013). The role of self-regulation in predicting sleep hygiene in university students. Psychol. Health Med..

[33] Srigley J.A., Corace K., Hargadon D.P., Yu D., MacDonald T., Fabrigar L., Garber G. (2015). Applying psychological frameworks of behaviour change to improve healthcare worker hand hygiene: A systematic review. J. Hosp. Infect..

[34] Peach H.D., Gaultney J.F., Ruggiero A.R. (2018). Direct and Indirect Associations of Sleep Knowledge and Attitudes with Objective and Subjective Sleep Duration and Quality via Sleep Hygiene. J. Prim. Prev..

[35] Hagger M.S., Chan D.K., Protogerou C., Chatzisarantis N.L. (2016). Using meta-analytic path analysis to test theoretical predictions in health behavior: An illustration based on meta-analyses of the theory of planned behavior. Prev. Med..

[36] McEachan R.R.C., Conner M., Taylor N.J., Lawton R.J. (2011). Prospective prediction of health-related behaviours with the theory of planned behaviour: A meta-analysis. Health Psychol. Rev..

[37] Jenner E.A., Watson P.W.B., Miller L., Jones F., Scott G.M. (2002). Explaining hand hygiene practice: An extended application of the Theory of Planned Behaviour. Psychol. Health Med..

[38] O’Boyle C.A., Henly S.J., Larson E. (2001). Understanding adherence to hand hygiene recommendations: The theory of planned behavior. Am. J. Infect. Control..

[39] Jeong S.Y., Kim K.M. (2016). Influencing factors on hand hygiene behavior of nursing students based on theory of planned behavior: A descriptive survey study. Nurse Educ. Today.

[40] Strong C., Lin C.Y., Jalilolghadr S., Updegraff J.A., Broström A., Pakpour A.H. (2018). Sleep hygiene behaviours in Iranian adolescents: An application of the Theory of Planned Behavior. J. Sleep Res..

[41] Knowlden A.P., Sharma M., Bernard A.L. (2012). A theory of planned behavior research model for predicting the sleep intentions and behaviors of undergraduate college students. J. Prim. Prev..

[42] Lao H.C., Tao V.Y., Wu A.M. (2016). Theory of planned behaviour and healthy sleep of college students. Aust. J. Psychol..

[43] Hagger M.S., Luszczynska A. (2014). Implementation intention and action planning interventions in health contexts: State of the research and proposals for the way forward. Appl. Psychol. Health Well Being.

[44] Zhang C.-Q., Zhang R., Schwarzer R., Hagger M.S. (2019). A meta-analysis of the health action process approach. Health Psychol..

[45] Reyes Fernández B., Knoll N., Hamilton K., Schwarzer R. (2016). Social-cognitive antecedents of hand washing: Action control bridges the planning–behaviour gap. Psychol. Health.

[46] Chow S., Mullan B. (2010). Predicting food hygiene. An investigation of social factors and past behaviour in an extended model of the Health Action Process Approach. Appetite.

[47] Hamilton K., Ng H., Zhang C.-Q., Phipps D., Zhang R. (2020). Social psychological predictors of sleep hygiene behaviors in Australian and Hong Kong university students. Int. J. Behav. Med..

[48] Nisbett R.E., Peng K., Choi I., Norenzayan A. (2001). Culture and systems of thought: Holistic versus analytic cognition. Psychol. Rev..

[49] Gollwitzer P.M., Sheeran P. (2006). Implementation intentions and goal achievement: A meta-analysis of effects and processes. Adv. Exp. Soc. Psychol..

[50] Zhang C.-Q., Wong M.C.Y., Zhang R., Hamilton K., Hagger M.S. (2019). Adolescent sugar-sweetened beverage consumption: An extended Health Action Process Approach. Appetite.

[51] Hagger M.S., Polet J., Lintunen T. (2018). The reasoned action approach applied to health behavior: Role of past behavior and tests of some key moderators using meta-analytic structural equation modeling. Soc. Sci. Med..

[52] World Health Organization Hand Hygiene: Why, How & When?. https://www.who.int/gpsc/5may/Hand_Hygiene_Why_How_and_When_Brochure.pdf.

[53] National Sleep Foundation Sleep Hygiene. https://www.sleepfoundation.org/articles/sleep-hygiene.

[54] Sniehotta F.F., Schwarzer R., Scholz U., Schuz B. (2005). Action planning and coping planning for long-term lifestyle change: Theory and assessment. Eur. J. Soc. Psychol..

[55] Kock N. WarpPLS User Manual: Version 6.0. http://cits.tamiu.edu/WarpPLS/UserManual_v_6_0.pdf.

[56] Tenenhaus M., Vinzi V.E., Chatelin Y.M., Lauro C. (2005). PLS path modeling. Compu. Sta. Data Analy..

[57] Ajzen I. Behavioral Interventions based on the Theory of Planned Behavior. http://people.umass.edu/aizen/pdf/tpb.intervention.pdf..

[58] De Bruijn G.J., Rhodes R.E., van Osch L. (2012). Does action planning moderate the intention-habit interaction in the exercise domain? A three-way interaction analysis investigation. J. Behav. Med..

[59] Van Osch L., Beenackers M., Reubsaet A., Lechner L., Candel M., de Vries H. (2009). Action planning as predictor of health protective and health risk behavior: An investigation of fruit and snack consumption. Int. J. Behav. Nutr. Phys. Act..

[60] Hagger M.S. (2019). Habit and physical activity: Theoretical advances, practical implications, and agenda for future research. Psychol. Sport Exerc..

[61] Rhodes R.E., Yao C.A. (2015). Models accounting for intention-behavior discordance in the physical activity domain: A user’s guide, content overview, and review of current evidence. Int. J. Behav. Nutr. Phys. Act..

[62] Wood W., Rünger D. (2016). Psychology of habit. Annu. Rev. Psychol..

[63] Ouellette J.A., Wood W. (1998). Habit and intention in everyday life: The multiple processes by which past behavior predicts future behavior. Psychol. Bull..

[64] Verplanken B., Orbell S. (2003). Reflections on past behavior: A self-report index of habit strength. J. Appl. Soc. Psychol..

[65] Van Bree R.J.H., van Stralen M.M., Mudde A.N., Bolman C., de Vries H., Lechner L. (2015). Habit as mediator of the relationship between prior and later physical activity: A longitudinal study in older adults. Psychol. Sport Exerc..

[66] Rothman A.J., Klein W.M.P., Sheeran P., Hagger M.S., Cameron L., Hamilton K., Hankonen N., Lintunen T. (2020). Moving from theoretical principles to intervention strategies: Applying the experimental medicine approach. The Handbook of Behavior Change.

[67] Hagger M.S., Cameron L.D., Hamilton K., Hankonen N., Lintunen T., Hagger M.S., Cameron L., Hamilton K., Hankonen N., Lintunen T. (2020). The science of behavior change: The road ahead. The Handbook of Behavior Change.

[68] Shiffman S., Stone A.A. (1998). Introduction to the special section: Ecological momentary assessment in health psychology. Health Psychol..

[69] Downs D.S., Hausenblas H.A. (2005). Elicitation studies and the theory of planned behavior: A systematic review of exercise beliefs. Psychol. Sport Exerc..

[70] Ajzen I. Constructing a Theory of Planned Behavior Questionnaire. https://people.umass.edu/aizen/pdf/tpb.measurement.pdf.

