# Supplementary material for: Predicting Hand Washing and Sleep Hygiene Behaviors among College Students: Test of an Integrated Social-Cognition Model
Source: Int J Environ Res Public Health. 2020 Feb 13;17(4):1209. doi: 10.3390/ijerph17041209 (PMC7068472; doi:10.3390/ijerph17041209)
Supplement: Supplementary file 1 [file ijerph-17-01209-s001.pdf]

Table S1

Standardized Parameter Estimates for the Direct, Indirect, and Total Effects in the Integrated Model of Hand Washing Excluding Past Behavior

Effect	β	*CI*$_{95}$		*ES*
		LL	UL	
Direct effects				
ASE → Intention	.026	-.087	.140	.008
Attitudes → Intention	.152**	.041	.263	.063
SN → Intention	.323***	.215	.431	.182
PBC → Intention	.360***	.252	.467	.209
PBC → Hand Washing	.022	-.091	.136	.004
ASE → MSE	.215***	.105	.325	.047
MSE → AP	.438***	.331	.544	.198
MSE → CP	.506***	.401	.611	.264
MSE → Hand Washing	.133*	.022	.245	.037
Intention → AP	.080	-.033	.192	.014
Intention → CP	−.066	-.178	.047	.010
Intention → Hand Washing	.120*	.008	.231	.024
AP → Hand Washing	.142**	.030	.253	.040
CP → Hand Washing	.099*	-.013	.211	.027
AP x Intention → Hand Washing	−.190***	-.301	-.080	.040
Age → ASE	.079	-.034	.191	.006
Age → Attitudes	.040	-.073	.153	.002
Age → SN	.048	-.065	.161	.003
Age → PBC	−.077	-.190	.035	.007
Age → Intention	.101	-.011	.213	.008
Age → MSE	-.028	-.141	.086	.001
Age → AP	-.126*	-.238	-.015	.016
Age → CP	.124*	.013	.236	.016
Age → Hand Washing	-.013	-.126	.101	.001
Gender → ASE	.030	-.083	.144	.001
Gender → Attitudes	.065	-.047	.178	.004
Gender → SN	.146**	.035	.257	.022
Gender → PBC	.078	-.035	.190	.007
Gender → Intention	-.003	-.117	.110	.000
Gender → MSE	.050	-.063	.163	.003
Gender → AP	-.047	-.160	.066	.000
Gender → CP	.030	-.083	.143	.002

Gender → Hand Washing	.207***	.097	.317	.049
Indirect effects				
ASE → Intention, MSE → AP	.096*	-.016	.208	.014
ASE → Intention, MSE → CP	.107*	-.005	.219	.005
ASE → Intention, MSE → Hand Washing	.032	-.082	.146	.002
ASE → Intention, MSE → AP, CP → Hand Washing	.024	-.090	.138	.002
Attitudes → Intention → AP	.012	-.068	.092	.002
Attitudes → Intention → CP	-.010	-.090	.070	.001
Attitudes → Intention → Hand Washing	.018	-.062	.098	.004
Attitudes → Intention → AP, CP → Hand Washing	.001	-.091	.093	.000
SN → Intention → AP	.026	-.054	.106	.004
SN → Intention → CP	-.021	-.101	.059	.003
SN → Intention → Hand Washing	.039	-.041	.119	.007
SN → Intention → AP, CP → Hand Washing	.002	-.090	.094	.000
PBC → Intention → AP	.029	-.051	.109	.006
PBC → Intention → CP	-.024	-.104	.056	.003
PBC → Intention → Hand Washing	.043	-.037	.123	.008
PBC → Intention → AP, CP → Hand Washing	.002	-.090	.094	.000
Intention → AP, CP → Hand Washing	.005	-.108	.119	.001
MSE → AP, CP → Hand Washing	.112*	.000	.224	.031
Total effects				
ASE → Hand Washing	.056	-.058	.170	.004
Attitudes → Hand Washing	.019	-.073	.111	.004
SN → Hand Washing	.040	-.052	.132	.007
PBC → Hand Washing	.067	-.044	.179	.012
Intention → Hand Washing	.124*	.012	.236	.025
MSE → Hand Washing	.245***	.135	.355	.069

Note. ASE = Action self-efficacy; SN = Subjective norm; PBC = Perceived behavioral control; MSE = Maintenance self-efficacy; AP = Action planning; CP = Coping planning. β = Standardized path coefficient; CI_{95} = 95% confidence interval of path coefficient; ES = Effect size estimate; LL = lower limit & UL = upper limit. $^{*}p < .05$ $^{**}p < .01$ $^{***}p < .001$

Table S2

Standardized Parameter Estimates for the Direct, Indirect, and Total Effects in the Integrated Model of Hand Washing Including Past Behavior

Effect	*β*	CI_{95}		*ES*
		LL	UL	
Direct effects				
PB → ASE	.212***	.102	.322	.045
PB → Attitudes	.308***	.199	.416	.096
PB → SN	.297***	.188	.405	.091
PB → PBC	.347***	.239	.455	.123
PB → Intention	.027	-.087	.140	.007
PB → MSE	.193***	.082	.303	.046
PB → AP	.185***	.074	.295	.053
PB → CP	.066	-.046	.179	.012
PB → Hand Washing	.216***	.106	.326	.071
Age → ASE	.078	-.035	.190	.006
Age → Attitudes	.017	-.096	.131	.001
Age → SN	.036	-.078	.149	.002
Age → PBC	-.048	-.161	.065	.004
Age → Intention	.100*	-.012	.212	.008
Age → MSE	.035	-.079	.148	.001
Age → AP	-.116*	-.228	-.005	.015
Age → CP	.122*	.010	.234	.016
Age → Hand Washing	-.014	-.127	.100	.001
Gender → ASE	.015	-.099	.128	.000
Gender → Attitudes	.053	-.060	.165	.003
Gender → SN	.128*	.017	.240	.019
Gender → PBC	.062	-.050	.175	.005
Gender → Intention	-.004	-.117	.110	.000
Gender → MSE	.039	-.074	.152	.003
Gender → AP	-.050	-.163	.063	.000
Gender → CP	.029	-.085	.142	.002
Gender → Hand Washing	.203***	.093	.314	.048
ASE → Intention	.031	-.083	.144	.009
Attitudes → Intention	.157**	.046	.268	.065
SN → Intention	.327***	.219	.435	.184
PBC → Intention	.363***	.256	.471	.211
PBC → Hand Washing	.039	-.074	.152	.007

ASE → MSE	.166**	.056	.277	.036
MSE → AP	.404***	.297	.511	.182
MSE → CP	.495***	.390	.600	.258
MSE → Hand Washing	.124*	.013	.236	.035
Intention → AP	.051	-.062	.164	.009
Intention → CP	-.058	-.171	.054	.009
Intention → Hand Washing	.108*	-.004	.220	.022
AP → Hand Washing	.112*	.000	.224	.032
CP → Hand Washing	.091	-.022	.203	.025
AP x Intention → Hand Washing	-.165**	-.276	-.055	.035
Indirect effects				
PB → ASE, Attitudes, SN, PBC → Intention	.278***	.168	.388	.070
PB → ASE → MSE	.035	-.045	.15	.008
PB → Intention, MSE → AP	.079	-.032	.191	.023
PB → Intention, MSE → CP	.094	-.018	.206	.017
PB → PBC, Intention, MSE, AP, CP → Hand Washing	.067	-.045	.179	.022
PB → ASE, Attitudes, SN, PBC → Intention → AP	.028	-.086	.142	.008
PB → ASE, Attitudes, SN, PBC → Intention → CP	.001	-.113	.115	.000
PB → Intention, MSE → AP, CP → Hand Washing & PB → ASE, Attitudes, SN, PBC → Intention → Hand Washing	.052	-.062	.166	.017
PB→ASE, Attitudes, SN, PBC→ Intention, MSE→AP, CP→ Hand Washing	.003	-.111	.117	.001
ASE → Intention, MSE → AP	.069	-.043	.181	.010
ASE → Intention, MSE → CP	.081	-.031	.193	.004
ASE → Intention, MSE → Hand Washing	.024	-.090	.138	.002
ASE → Intention, MSE → AP, CP → Hand Washing	.015	-.099	.129	.000
Attitudes → Intention → AP	.008	-.072	.088	.001
Attitudes → Intention → CP	-.009	-.089	.071	.001
Attitudes → Intention → Hand Washing	.017	-.063	.097	.003
Attitudes → Intention → AP, CP → Hand Washing	.000	-.092	.092	.000
SN → Intention → AP	.017	-.063	.097	.003
SN → Intention → CP	-.019	-.099	.061	.002
SN → Intention → Hand Washing	.035	-.045	.115	.006
SN → Intention → AP, CP → Hand Washing	.000	-.092	.092	.000
PBC → Intention → AP	.019	-.061	.099	.004
PBC → Intention → CP	-.021	-.101	.059	.003
PBC → Intention → Hand Washing	.039	-.041	.119	.007
PBC → Intention → AP, CP → Hand Washing	.000	-.092	.092	.000
Intention → AP, CP → Hand Washing	.000	-.113	.114	.000
MSE → AP, CP → Hand Washing	.090	-.022	.202	.025

Total effects				
ASE → Hand Washing	.039	-.075	.153	.002
Attitudes → Hand Washing	.017	-.075	.109	.003
SN → Hand Washing	.035	-.057	.127	.006
PBC → Hand Washing	.078	-.034	.190	.014
Intention → Hand Washing	.109*	-.003	.221	.022
MSE → Hand Washing	.215***	.105	.325	.060
PB → Hand Washing	.338***	.230	.446	.111

Note. ASE = Action self-efficacy; SN = Subjective Norm; PBC = Perceived behavioral control; MSE = Maintenance self-efficacy; AP = Action planning; CP = Coping planning; PB = Past behavior. β = Standardized path coefficient; CI_{95} = 95% confidence interval of path coefficient; ES = Effect size estimate; LL = lower limit & UL = upper limit. $^{*}p < .05$ $^{**}p < .01$ $^{***}p < .001$

Table S3

Standardized Parameter Estimates for the Direct, Indirect, and Total Effects in the Integrated Model of Sleep Hygiene Excluding Past Behavior

Effect	β	*CI*95		*ES*
		LL	UL	
Direct effects				
ASE → Intention	.062	-.051	.175	.013
Attitudes → Intention	.162**	.051	.272	.050
SN → Intention	.314***	.206	.423	.143
PBC → Intention	.286***	.177	.395	.134
PBC → Sleep Hygiene	.120*	.008	.231	.034
ASE → MSE	.147**	.036	.258	.021
MSE → AP	.365***	.253	.468	.135
MSE → CP	.285***	.176	.393	.082
MSE → Sleep Hygiene	.036	-.077	.149	.005
Intention → AP	.096*	-.016	.208	.016
Intention → CP	-.083	-.195	.029	.008
Intention → Sleep Hygiene	.158**	.048	.269	.042
AP → Sleep Hygiene	.237***	.127	.346	.075
CP → Sleep Hygiene	.132*	.021	.244	.027
AP x Intention → Sleep Hygiene	-.079	-.192	.033	.018
Age → ASE	.054	-.059	.167	.003
Age → Attitudes	-.063	-.176	.049	.004
Age → SN	.063	-.049	.176	.003
Age → PBC	.032	-.081	.145	.001
Age → Intention	.100*	-.012	.212	.010
Age → MSE	.073	-.039	.186	.005
Age → AP	-.150**	-.261	-.039	.020
Age → CP	.050	-.063	.163	.003
Age → Sleep Hygiene	-.056	-.169	.057	.004
Gender → ASE	-.007	-.121	.106	.000
Gender → Attitudes	.103*	-.008	.215	.011
Gender → SN	.185***	.074	.295	.034
Gender → PBC	.081	-.031	.193	.006
Gender → Intention	.030	-.083	.143	.004
Gender → MSE	.098*	-.014	.210	.008
Gender → AP	-.062	-.175	.051	.001
Gender → CP	-.098*	-.210	.014	.008

Gender → Sleep Hygiene	.157**	.047	.268	.029
Indirect effects				
ASE → Intention, MSE → AP	.059	-.053	.171	.011
ASE → Intention, MSE → CP	.037	-.077	.151	.002
ASE → Intention, MSE → Sleep Hygiene	.015	-.099	.129	.001
ASE → Intention, MSE → AP, CP → Sleep Hygiene	.019	-.095	.132	.001
Attitudes → Intention → AP	.015	-.065	.095	.002
Attitudes → Intention → CP	-.013	-.093	.067	.000
Attitudes → Intention → Sleep Hygiene	.026	-.054	.106	.006
Attitudes → Intention → AP, CP → Sleep Hygiene	.002	-.090	.094	.000
SN → Intention → AP	.030	-.050	.110	.003
SN → Intention → CP	-.026	-.106	.054	.000
SN → Intention → Sleep Hygiene	.050	-.030	.130	.010
SN → Intention → AP, CP → Sleep Hygiene	.004	-.088	.096	.001
PBC → Intention → AP	.027	-.053	.107	.006
PBC → Intention → CP	-.024	-.104	.056	.003
PBC → Intention → Sleep Hygiene	.045	-.035	.125	.013
PBC → Intention → AP, CP → Sleep Hygiene	.003	-.089	.095	.001
Intention → AP, CP → Sleep Hygiene	.012	-.102	.126	.003
MSE → AP, CP → Sleep Hygiene	.123*	.011	.235	.017
Total effects				
ASE → Sleep Hygiene	.034	-.080	.148	.003
Attitudes → Sleep Hygiene	.028	-.064	.120	.006
SN → Sleep Hygiene	.054	-.038	.146	.010
PBC → Sleep Hygiene	.169**	.057	.281	.048
Intention → Sleep Hygiene	.170**	.060	.280	.045
MSE → Sleep Hygiene	.159**	.047	.271	.022

Note. ASE = Action self-efficacy; SN = Subjective norm; PBC = Perceived behavioral control; MSE = Maintenance self-efficacy; AP = Action planning; CP = Coping planning. β = Standardized path coefficient; CI_{95} = 95% confidence interval of path coefficient; ES = Effect size estimate; LL = lower limit & UL = upper limit. $^{*}p < .05$ $^{**}p < .01$ $^{***}p < .001$

Table S4

Standardized Parameter Estimates for the Direct, Indirect, and Total Effects in the Integrated Model of Sleep Hygiene Including Past Behavior

Effect	*β*	*CI*₉₅ LL	*CI*₉₅ UL	*ES*
Direct effects				
PB → ASE	.085	-.027	.197	.007
PB → Attitudes	.220***	.110	.330	.048
PB → SN	.201***	.091	.312	.042
PB → PBC	.460***	.354	.566	.212
PB → Intention	.078	-.034	.190	.022
PB → MSE	.070	-.042	.183	.007
PB → AP	.197***	.087	.307	.050
PB → CP	.085	-.027	.197	.011
PB → Sleep Hygiene	.187***	.077	.298	.060
Age → ASE	.054	-.059	.167	.003
Age → Attitudes	-.056	-.169	.057	.003
Age → SN	.060	-.052	.173	.003
Age → PBC	.009	-.105	.122	.000
Age → Intention	.102	-.010	.214	.010
Age → MSE	.060	-.053	.172	.004
Age → AP	-.131*	-.243	-.020	.017
Age → CP	.039	-.074	.152	.003
Age → Sleep Hygiene	-.068	-.181	.044	.005
Gender → ASE	-.004	-.118	.110	.000
Gender → Attitudes	.115*	.004	.227	.012
Gender → SN	.179***	.069	.290	.033
Gender → PBC	.074	-.038	.187	.006
Gender → Intention	.035	-.078	.148	.005
Gender → MSE	.095*	-.017	.207	.008
Gender → AP	-.054	-.167	.058	.001
Gender → CP	-.097*	-.209	.015	.008
Gender → Sleep Hygiene	.159**	.049	.270	.030
ASE → Intention	.066	-.046	.179	.014
Attitudes → Intention	.155**	.044	.266	.048
SN → Intention	.313***	.205	.421	.143
PBC → Intention	.253***	.143	.362	.118
PBC → Sleep Hygiene	.045	-.068	.158	.013

ASE → MSE	.143**	.031	.254	.021
MSE → AP	.349***	.242	.457	.131
MSE → CP	.279***	.170	.388	.081
MSE → Sleep Hygiene	.017	-.096	.130	.002
Intention → AP	.058	-.055	.171	.010
Intention → CP	-.070	-.183	.042	.007
Intention → Sleep Hygiene	.146**	.034	.257	.039
AP → Sleep Hygiene	.208***	.098	.318	.066
CP → Sleep Hygiene	.118*	.006	.230	.024
AP x Intention → Sleep Hygiene	-.077	-.189	.035	.018
Indirect effects				
PB → ASE, Attitudes, SN, PBC → Intention	.219***	.109	.329	.061
PB → ASE → MSE	.012	-.068	.092	.001
PB → Intention, MSE → AP	.029	-.085	.143	.007
PB → Intention, MSE → CP	.014	-.100	.128	.002
PB → PBC, Intention, MSE, AP, CP → Sleep Hygiene	.084	-.028	.196	.027
PB → ASE, Attitudes, SN, PBC → Intention → AP	.017	-.097	.131	.004
PB → ASE, Attitudes, SN, PBC → Intention → CP	-.012	-.126	.102	.001
PB → Intention, MSE → AP, CP → Sleep Hygiene & PB → ASE, Attitudes, SN, PBC → Intention → Sleep Hygiene	.040	-.074	.154	.013
ASE → Intention, MSE → AP	.054	-.060	.168	.010
ASE → Intention, MSE → CP	.035	-.079	.149	.002
ASE → Intention, MSE → Sleep Hygiene	.012	-.102	.126	.001
ASE → Intention, MSE → AP, CP → Sleep Hygiene	.015	-.099	.129	.001
Attitudes → Intention → AP	.009	-.071	.089	.001
Attitudes → Intention → CP	-.011	-.091	.069	.000
Attitudes → Intention → Sleep Hygiene	.022	-.058	.102	.005
Attitudes → Intention → AP, CP → Sleep Hygiene	.001	-.091	.093	.000
SN → Intention → AP	.018	-.062	.098	.002
SN → Intention → CP	-.022	-.102	.058	.000
SN → Intention → Sleep Hygiene	.046	-.034	.126	.009
SN → Intention → AP, CP → Sleep Hygiene	.001	-.091	.093	.000
PBC → Intention → AP	.015	-.065	.095	.003
PBC → Intention → CP	-.018	-.098	.062	.003
PBC → Intention → Sleep Hygiene	.037	-.043	.117	.010
PBC → Intention → AP, CP → Sleep Hygiene	.001	-.091	.093	.000
Intention → AP, CP → Sleep Hygiene	.004	-.110	.118	.001
MSE → AP, CP → Sleep Hygiene	.106*	-.006	.218	.015
Total effects				

ASE → Sleep Hygiene	.027	-.087	.141	.002
Attitudes → Sleep Hygiene	.023	-.069	.115	.005
SN → Sleep Hygiene	.047	-.045	.139	.009
PBC → Sleep Hygiene	.083	-.029	.195	.024
Intention → Sleep Hygiene	.149**	.037	.261	.040
MSE → Sleep Hygiene	.123*	.011	.235	.017
PB → Sleep Hygiene	.314***	.206	.422	.100

Note. ASE = Action self-efficacy; SN = Subjective Norm; PBC = Perceived behavioral control; MSE = Maintenance self-efficacy; AP = Action planning; CP = Coping planning; PB = Past behavior. β = Standardized path coefficient; CI_{95} = 95% confidence interval of path coefficient; ES = Effect size estimate; LL = lower limit & UL = upper limit. $^{*}p < .05$ $^{**}p < .01$ $^{***}p < .001$
